# Patients’ perceived needs for medical services for non-specific low back pain: A systematic scoping review

**DOI:** 10.1371/journal.pone.0204885

**Published:** 2018-11-08

**Authors:** Louisa Chou, Tom A. Ranger, Waruna Peiris, Flavia M. Cicuttini, Donna M. Urquhart, Kaye Sullivan, Maheeka Seneviwickrama, Andrew M. Briggs, Anita E. Wluka

**Affiliations:** 1 Department of Epidemiology and Preventive Medicine, School of Public Health and Preventive Medicine, Monash University, Melbourne, Victoria, Australia; 2 Monash University Library, Monash University, Melbourne, Victoria, Australia; 3 School of Physiotherapy and Exercise Science, Curtin University, Perth, Australia; 4 MOVE: muscle, bone & joint health, Victoria, Australia; Brown University, UNITED STATES

## Abstract

**Background:**

An improved understanding of patients’ perceived needs for medical services for low back pain (LBP) will enable healthcare providers to better align service provision with patient expectations, thus improving patient and health care system outcomes. Thus, we aimed to identify the existing literature regarding patients’ perceived needs for medical services for LBP.

**Methods:**

A systematic scoping review was performed of publications identified from MEDLINE, EMBASE, CINAHL and PsycINFO (1990–2016). Descriptive data regarding each study, its design and methodology were extracted and risk of bias assessed. Aggregates of patients’ perceived needs for medical services for LBP were categorised.

**Results:**

50 studies (35 qualitative, 14 quantitative and 1 mixed-methods study) from 1829 were relevant. Four areas of perceived need emerged: (1) Patients with LBP sought healthcare from medical practitioners to obtain a diagnosis, receive management options, sickness certification and legitimation for their LBP. However, there was dissatisfaction with the cursory and superficial approach of care. (2) Patients had concerns about pharmacotherapy, with few studies reporting on patients’ preferences for medications. (3) Of the few studies which examined the patients’ perceived need of invasive therapies, these found that patients avoided injections and surgeries (4) Patients desired spinal imaging for diagnostic purposes and legitimation of symptoms.

**Conclusions:**

Across many different patient populations with data obtained from a variety of study designs, common themes emerged which highlighted areas of patient dissatisfaction with the medical management of LBP, in particular, the superficial approach to care perceived by patients and concerns regarding pharmacotherapy. Patients perceive unmet needs from medical services, including the need to obtain a diagnosis, the desire for pain control and the preference for spinal imaging. These issues need to be considered in developing approaches for the management of LBP in order to improve patient outcomes.

## Introduction

Low back pain (LBP) is the leading cause of disability worldwide[1]. It is highly prevalent and is associated with pain, functional impairment, long-term incapacity, work absenteeism and high utilisation of healthcare[1,2]. LBP is costly, amounting to an estimated $88billion in the United States in 2013, with medical services comprising a considerable proportion of the incurred expenditure[3]. Consequently, several guidelines have been developed to guide the different presentations of acute and chronic back pain management, to direct clinical practice and to rationalise health care resource utilisation appropriately[4–10]. These guidelines recommend, as relevant to pain duration, a thorough clinical evaluation to exclude serious spinal pathology, judicious use of radiology, patient education to support optimal self-management, exercise therapy, psychological therapies for some people, short-term use of prescription medications and spinal manipulation for pain relief[6–10]. However, the publication and dissemination of guidelines does not ensure their implementation[11,12] and previous studies have demonstrated poor uptake of guidelines for the management of LBP [13–17]. Instead, there has been a significant rise in opioid prescribing for LBP, with a resultant 660% increase in expenditure in the United States[18] and an increase in complications such as opioid dependence, addiction and mortality associated with overdose[19]. Spinal imaging has also been inappropriately utilised (overuse when not indicated and underuse when indicated) [20], which has further contributed to the growing financial burden of LBP, as well as other ramifications including additional investigations, referrals and potentially invasive procedures, that for most represent low-value care[8]. Furthermore, despite the recommendations for active rehabilitation such as exercise therapy for LBP, less than 50% of patients report being referred for active rehabilitation programs [17,21]. Collectively, these practices have contributed to unhelpful beliefs held by clinicians and the public concerning appropriate management of LBP, with calls for reframing how back pain is understood and managed [22].

Clinical practice guidelines face multiple impediments to implementation. Barriers to execution include environmental factors, such as resource allocation and costs, as well as clinician-related barriers, including a lack of agreement with clinical practice guidelines, lack of awareness and familiarity with recommendations[16,23]. Patient factors are also critical to the successful uptake and adherence to guidelines[16,17]. Clinicians have reported that patients’ preferences are an important cause of non-adherence to guidelines[17]. Patients’ non-adherence may be related to the high level of patient dissatisfaction with LBP management from medical practitioners[24,25], which has historically focused on a biomedical model of care. This biomedical approach is typically based on the scientific academic literature conducted by healthcare professionals. However this approach may be flawed as it neither adequately takes into account the patient perspective, nor satisfactorily consider the psychological and social drivers to the pain experience[26]. Although there are previous reviews summarising the evidence regarding patient expectations and experiences of healthcare for LBP, none of these have focussed on the patients’ perceived needs for medical services[26,27]. Therefore, we aimed to review the existing literature regarding patients’ perceived needs for medical services for LBP.

## Methods

A systematic scoping review, based on the framework proposed by Arksey and O’Malley, was performed to enable a comprehensive exploration of the patients’ perspective[28]. Systematic scoping reviews are aimed at mapping key concepts, identifying gaps in the evidence, and reviewing different types of evidence[29,30]. This review was conducted within a larger project examining the patients’ perceived needs relating to musculoskeletal health[31]. The study methodology is similar to a previously published review examining patients’ perceived needs of health services for osteoarthritis[32].

### Search strategy and study selection

The literature search was performed by electronically searching relevant databases (MEDLINE, EMBASE, CINAHL and PsycINFO) between January 1990 and June 2016. This time period was chosen to include relevant studies examining the current patient perspective. The search strategy (see S1 File for full OVID Medline search strategy) was developed by one of the study investigators (MS), with input from clinician researchers (Rheumatologists, FC and AW and Physiotherapist, AB), a patient representative and an academic librarian (KL). The strategy combined both MeSH terms and text words to capture information regarding patients’ perceived needs for medical services for LBP (S3 Supplementary Appendix). We have used the term “medical services” to include any service provided by medical practitioners, including general practitioners, specialist physicians and surgeons. A broad definition was used for “patient perceived needs”, which referred to patients’ perception of services that provided them with the capacity to benefit, including their expectations of satisfaction and preferences for medical services[33]. LBP was defined as non-specific LBP, with or without leg pain, excluding back pain from fractures, malignancy, infection and inflammatory spinal disorders.

Two reviewers, including LC and one of LC, TR and WP independently assessed the titles and abstracts of all studies identified by the initial search for relevance. Discrepancies in the inclusion of studies were reviewed by a third investigator (AW) to reach consensus. The initial screening was set to be open-ended to retain as many relevant studies as possible, with no restriction on the study methods. Studies were included if they met the following criteria: (1) included patients older than 18 years, (2) recruited patients with non-specific LBP and (3) reported on patients’ perceived needs for medical services for LBP. Studies were limited to human studies in the English language and full-text articles. No restrictions were applied to the prevalence of LBP and studies concerning acute, subacute and chronic LBP were included. Those that appeared to meet inclusion criteria were retrieved and the full text was assessed for relevance (LC). The reference lists of identified studies and review articles were searched to find possible further studies for inclusion.

### Data extraction and analysis

The following data were systematically extracted by one investigator (LC) using a data extraction form specifically developed for this review: (1) primary study aim, (2) study population (patient age and gender, population source, population size and definition of LBP), (3) description of the study methods and (4) year of publication. Included studies were reviewed to identify aspects of medical services that patients had a preference for, expected, or were satisfied with using principles of meta-ethnography to synthesise qualitative data[34]. One author (LC) developed a framework of concepts and underlying themes, based on primary data in the included studies. Reciprocal translational analysis[34] was then undertaken to identify key concepts from individual studies and then translating and comparing these concepts to other studies to gradually explore and map the overarching themes. Data was extracted based on a customised data collection form. The framework of concepts and underlying themes were independently reviewed by three senior authors (FC and AW with over 15 years of clinical rheumatology consultant-level experience and a senior physiotherapist, AMB) to ensure accuracy of the extracted data and clinical meaningfulness.

### Methodological quality assessment

To assess the methodological quality of the included studies, two from a panel of three (LC, TR, WP) independently assessed the methodological quality of all included studies.

Qualitative studies were assessed using the Critical Appraisal Skills Programme (CASP) tool[35]. The CASP is commonly used to assess qualitative research studies[35]. This tool has 10 questions that assists readers appraise articles based on appropriate research design (CASP questions 2–3), sampling (CASP question 4), data collection (CASP question 5), bias (CASP question 6), ethical issues (CASP question 7), data analysis (CASP question 8), research findings (CASP question 9) and the value of the research (CASP question 10). Each question is scored ‘yes’, ‘no’ or ‘cannot tell’ regarding the study quality and potential for bias. This is no overall score for the level of bias.

Hoy’s risk of bias tool was utilised to assess the external and internal validity of quantitative studies. This tool was developed to examine study quality and risk of bias in prevalence studies. This tool is comprised of 10 questions that assess the external validity (questions 1–4) and internal validity (questions 5–10) of a study. Each question is scored either ‘yes’ (low risk of bias) or ‘no’ (high risk of bias). Thus for a study to be determined to be at a low risk of bias it was defined as scoring 8 or more “yes” answers, moderate risk of bias was defined as 6 to 7 “yes” answers and high risk of bias was defined as 5 or fewer “yes” answers[36]. Disagreements were resolved initially through consensus, with remaining conflicts reviewed by the senior author (AW).

## Results

### Overview of articles

**Fig 1:**
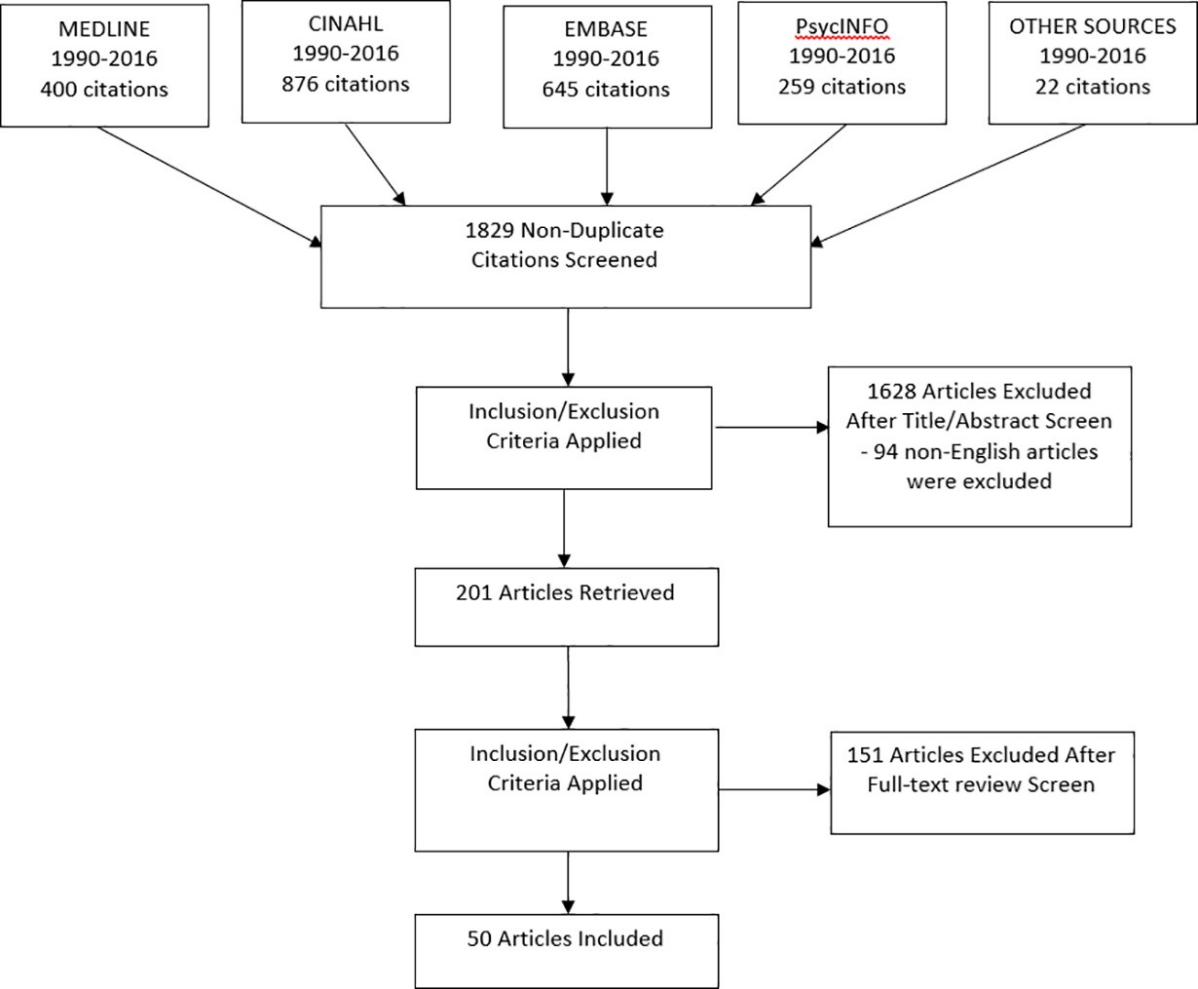
Modified PRISMA flow diagram.

The search returned 1829 articles, of which 50 studies explored LBP patients’ perceived needs for medical services (Table 1). A PRISMA flow diagram detailing the study selection is shown in Fig 1. The descriptive characteristics of the included studies are shown in Table 1. Of these, 19 were from the United Kingdom[24,25,37–54], 13 from the United States of America[55–66], 9 from Europe[67–75], 8 from Australasia[76–83] and one from the Middle-East[84].

**Table 1:** Studies identified in the systematic review of patients’ perceived needs for medical services for low back pain.

Author, year & country	Diagnosis of back pain	Participants	Source of participants	Age and gender	Primary Study Aim	Study design & data collection
Allegretti[66] 2010 USA	Chronic LBP (>6 months of daily or near daily pain)	23 participants	Purposeful sample from Family Care Centre, Memorial Hospital.	Average age 45 (28–72) 52% female	To explore discrepancies between patients with chronic LBP and physicians using paired interviews of shared experiences	Qualitative: In depth interviews
Amonkar[24] 2011 UK	Duration of LBP not specified 46.2% of men had a history of LBP and 49.4% of women had a history of LBP	81 GPs and 533 patients participated	50 consecutive patients were recruited from 12 GP practices.	Age distribution not specified 63% Female	To investigate whether doctors and patients have different perceptions and expectations with respect to the management of simple chronic back pain.	Quantitative Questionnaires
Banbury[54] 2008 UK	LBP for >6 weeks	16 participants	Convenience sample of patients referred to the Nottingham Back Team by their GP	Age range 18–65 31% female	To explore the attitudes and experiences of analgesic use of patients with LBP and referred to a back pain program.	Qualitative Semi-structured interview
Borkan[84] 1995 Israel	At least 1 episode of LBP (patients not included on basis of intensity/duration of pain) Duration of LBP not specified	66 participants	10 focus groups, 3 geographic locations from family medicine practices. Participants were identified by community nurses, physicians or through chart review (purposive recruitment).	Average age 39.5 (range 18–67) 35% female	To increase the understanding of low back pain through access to patients’ perceptions, beliefs, illness behaviours and lived experiences.	Qualitative Focus groups, individual interviews and participant observation
Buchbinder[65] 2015 USA	Duration of LBP not specified	32 doctors and 74 patients participated	Participants were identified using the electronic medical record.	Age distribution not specified. 50% female	To examine requests for analgesia among patients presenting with back pain to ED	Qualitative Audio-recording of encounters
Campbell[53] 2007 UK	LBP > 1 year	16 participants	Patients who completed a Pain Management Program and requested further secondary care for continuing pain.	Age range 34–78 Gender of patients not specified	To examine expectations for pain treatment and outcome To determine whether they are influential in maintaining health service consumption	Qualitative Group discussions
Carey[63] 1995 USA	LBP <10 weeks duration	1555 participants	208 practitioners in North Carolina, randomly selected from 6 strata (urban primary care physicians, rural primary care physicians, urban chiropractors, rural chiropractors, orthopaedic surgeons and primary care providers) and asked to enrol consecutive patients with acute low back pain.	- Urban primary care physician: mean age 41, 66% female - Rural primary care physician: mean age 43, 57% female - Urban chiropractor: mean age 40, 50% female - Rural chiropractor: mean age 44, 45% female - Orthopaedics: mean age 40, 48% female - Health maintenance organisation: mean age 38, 58% female	To determine whether the outcomes of any charges for care differ among primary care practitioners, chiropractors and orthopaedic surgeons.	Quantitative Interviews and telephone surveys
Carey[62] 1996 USA	Severe low back pain–ie back pain leading the respondent unable to perform usual daily activities LBP (functionally limiting pain < 3 months)	485 participants	Participants with low back pain were recruited by stratified sampling of telephone numbers.	Patients seeing doctors: 19% of patients were > 60yo and 64% female Patients seeing chiropractors: 5% were >60yo and 27% female	To examine correlates of care-seeking in people with low back pain	Quantitative Telephone interviews
Carey[64] 1999 USA	Recurrence of back pain	208 GPs participants 754 patients	Practitioners randomly selected from medical and chiropractic state licensure files from 6 strata (see above study in 1995) [63]. Practitioners invited sequential patients with acute low back pain to participate.	Mean age 41.7 51% female	To explore the relationship between type of initial care as well as the likelihood of recurrence and consequent care seeking behaviour	Quantitative Telephone interviews
Chenot[73] 2007 Germany	Acute LBP = <90 days, recurrent LBP = multiple episodes of LBP of <90 days duration within the last 12 months, chronic LBP more than 90 consecutive days of LBP within the last 12 months	116 general practices and 1342 patients participated	Prospective cohort study embedded within a 3-armed RCT with an educational intervention in primary care. Consecutive patients with LBP recruited by general practitioners.	No specialist consultation: 35% age < 40yo, 43% age 40–60, 22% age > 60yo. 46% female. Specialist consultation: 28% age <40yo, 47% age 40–60, 25% age >60yo. 54% female	To explore (1) factors associated with LBP patients’ seeking specialist care and its appropriateness, (2) how specialist care affects management of LBP and (3) whether health care resources are over or under utilised	Quantitative Questionnaires and telephone interviews
Chew[52] 1997 UK	Back pain for more than 6 weeks in the previous year	20 participants	20 patients from a back pain clinic in Manchester were invited to participate.	Age range 21–56 55% female	To explore how sufferers of chronic LBP describe their pain and its impact on their lives and how their problem is dealt with their family doctor	Qualitative Semi-structured interviews
Cook[49] 2000 UK	Duration of LBP 6 months to 21 years	7 participants	7 patients were selected by the researcher who had attended the back rehabilitation program in the last 6 months	Age range 22–53 57% female	To explore how individual patients experienced LBP, their experience of active rehabilitation, and their perception of its’ influence of their subsequent ability to manage their problem.	Qualitative Semi-structured in-depth interviews which were audio-taped
Coole[51] 2010 UK	Duration of LBP not defined Mean LBP 6.8 years	25 participants	Participants were recruited during routine back assessment following referral by their GP or other healthcare professionals	Average age 44.7 (range 22–58) 52% female	To explore the experiences of employed people with back pain and their perceptions of how GPs and other clinicians have addressed their work difficulties	Qualitative Thematic analysis of individual interviews
Coole[50] 2010 UK	Duration of LBP not defined	25 participants	Convenience sample of low back pain patients referred for multidisciplinary rehabilitation	Average age 44.7 (range 22–58) 52% female	To explore the individual experiences and perceptions of patients awaiting rehabilitation who were concerned about their ability to work because of persisting, or recurrent low back pain	Qualitative Thematic analysis of semi-structured interviews
Crowe[82] 2010 New Zealand	LBP > 12 weeks	64 participants	Community health newsletters and physiotherapy clinics.	Mean age 55.1 (SD13.2). 48% Female	To report on the self-management strategies of people with chronic low back pain and how their healthcare professionals perceived their role in facilitating self-management.	Qualitative Semi-structured interviews
Darlow[83] 2012 New Zealand	Acute LBP <6 weeks and chronic LBP > 3 months	12 participants (acute LBP) and 11 (chronic LBP)	Volunteers, recruited by advertisements in health care facilities and public spaces in 1 region of NZ. Respondents were screened by telephone.	Acute LBP–Age 36.2 (13.1) and 58% female Chronic LBP–age 45.6 (14.1), 64% female	To explore the formation and impact of attitudes and beliefs among people experiencing acute and chronic LBP	Qualitative Semi-structured interviews
Darlow[77] 2015 New Zealand	Acute LBP <6 weeks and chronic LBP > 3 months	12 participants (acute LBP) and 11 (chronic LBP	Purposive sampling of participants recruited via advertisements in a range of health care facilities and public spaces	Acute LBP–Age 36.2 (13.1) and 58% female Chronic LBP–age 45.6 (14.1), 64% female	To explore attitudes, beliefs and perceptions related to low back pain and analyse how these might influence the perceived threat associated with back pain	Qualitative Semi-structured interviews
Dima[48] 2013 UK	LBP (> 6 weeks)	75 participants	Patients who had recently consulted their family doctor or CAM practitioner for LBP and were members of a chronic pain patient support group.	Median age 62 (range 29–85) 64% female	To explore patient’s beliefs about LBP treatments	Qualitative Focus groups
Franz[55] 2015 USA	Duration of LBP not defined	121 participants	Surveys of all new patients referred to a single neurosurgeon for evaluation of spinal spondylosis	Average age 54 (SD 16) 47% female	To determine patients’ referred to a neurosurgery clinic for degenerative spinal disorders understanding of lumbar spondylosis diagnosis and treatment	Quantitative Survey
Heyduck[72] 2014 Germany	Chronic LBP with no disc surgery within the past 6 months	201 participants	Study participants were recruited from 4 rehabilitation centres	Mean age 54.09 (SD 11.37) 63% female	To (i) describe the illness and treatment beliefs of chronic LBP patients and (ii) to explore the relation of illness and treatment beliefs to individual, disease and interaction related variables.	Quantitative Questionnaires
Hoffman[81] 2013 Australia	LBP < 3 months	11 participants	Convenience sample from urban GP practice	Median age 57 (range 22–72) 91% female	To explore the expectations of the management of patients presenting to primary care with acute LBP	Qualitative Semi-structured telephone interview
Holt[38] 2015 UK	Duration of LBP not defined	23 participants	Patients recruited from GP surgeries in Northamptonshire	Average age 57.2 (SD 16) 44% female	To explore how patients with low back pain perceive practitioners’ reassuring behaviours during consultations	Qualitative Interviews
Jenkins[76] 2016 Australia	Duration of LBP not defined	300 participants	Consecutive patients attending medical practices were invited to participate	Mean age 44 (SD 18.9) 61% female	To investigate i) patient beliefs regarding the need for imaging in LBP and ii) whether personal characteristics, pain characteristics or back pain beliefs are associated with imaging beliefs	Quantitative Survey
Kawi[61] 2012 USA	Duration of LBP not specified	110 participants	Convenience sample of patients from Pain Centres.	Median age 47 (range 19–86) 59% female	To describe chronic LBP patients’ views to facilitate better understanding of their self-management, self-management support and functional ability.	Qualitative Surveys
Kirby[80] 2013 Australia	Women who had indicated in a survey that they sought help for back pain. Duration of LBP not specified	1310 participants	Sub-study of the Australian Longitudinal Study on Women’s Health. Women randomly selected from the national Medicare database and invited to participate.	Age range 59–64 100% female	To uncover and profile health care utilisation for back pain care and the actual out-of-pocket expenditure for a nationally representative sample of older Australian women	Quantitative Surveys and questionnaires
Klojgaard[70] 2014 Denmark	LBP > 2 months	348 participants	Data collected at the Spine Centre of Southern Denmark, the only public spine centre in the region	Mean age 54.65 (SD 0.73) 54% female	To increase the understanding of patients’ preferences regarding LBP treatment by quantifying the utilities and trade-offs of treatment options and treatment outcomes from the patient perspective.	Qualitative, quantitative and econometric analysis Questionnaire
Lacroix[75] 1995 Switzerland	Not reported	Not reported	Not reported	Not reported	“To show you the testimonies in order that the burden those patients have to carry because of their disease can be seen and heard in order to be better recognised”.	Qualitative Testimonials
Laerum[71] 2006 Norway	LBP > 3 months	35 patients	Purposive sampling of 35 consecutive patients with chronic low back pain referred to a specialist (11 specialists in neurology, rehabilitation medicine, orthopaedics, neurosurgery, rheumatology)–based on gender, age, duration of pain and education	Median age 45.5 (range 23–65) 49% female	To identify core elements of what patients with chronic low back pain perceive as good clinical communication and interaction with a specialist	Qualitative Patient interviews
Layzell[47] 2001 UK	Duration of LBP not specified	118 participants in group A and 12 in group B	Sample of patients treated for LBP by the physiotherapy department were mailed with a reply paid envelope (A) and 8 volunteers from the author’s workplace with a back problem and community volunteers (B)	Age distribution not specified Group A– 58% female and Group B– 50% female.	To assess patient satisfaction with the current services provided for back pain and to increase the level of understanding from the patients’ perspective on beliefs about their back pain and how it affects their daily life	Quantitative Questionnaires
Liddle[68] 2007 Ireland	Currently having or recently having LBP (non specific LBP) last 3 months or more and have received treatment within the previous 24 months	18 participants	Invitation by a campus-wide (University of Ulster) email, poster advertisement and word of mouth.	50% between with ages of 41-55yo 75% female	To explore the experiences, opinions and treatment expectations in chronic low back pain patients in order to identify what components of treatment they consider as being of most value	Qualitative Focus group interviews
Lyons[44] 2013 USA	LBP >1 year	48 participants	Recruitment by letter from patients’ lists at a family medicine clinic, chiropractic academic health centre and flyers at 2 senior centres and 3 senior housing sites.	Mean age 75.2 (SD 8) 79% female	To explore the perspectives of older adults toward LBP collaborative care by MDs (medical doctors) and DCs (doctor of chiropractic therapy)	Qualitative Focus group interviews
May[45] 2007 UK	Duration of LBP not specified	34 participants	Systematically sampled from patients who had received physiotherapy for low back pain from two physiotherapy departments in the UK.	Age range 29–77 59% female	To explore patients’ perspective and attitudes about back pain and it’s management using an explorative qualitative approach.	Qualitative Semi-structured interviews
McIntosh[46] 2003 UK	Consulted GP for LBP in the previous 12 months however duration of LBP not specified	15 GPs and 37 patients participated	Purposive sampling of 3 primary care centres.	Age and gender distribution not specified	To ascertain patients’ information needs from the perspectives of both patients and their GPs in order to suggest a suitable content for a patient information pack to be distributed to patients presenting in a primary care setting with acute low back pain	Qualitative Semi-structured interviews
McPhillips-Tangum[60] 1998 USA	People who had experienced low back pain during the 3 years preceding the study. Episodes were defined as >1 visits for LBP spaced at least 90 days apart from any other visit for LBP.	54 participants	Interviews were conducted in 3 cities (Atlanta, Dallas and Seattle). Computerised databases used to identify eligible participants. Random sample of 50 in Atlanta, 35 in Dallas and 25 in Seattle were invited to participate.	Mean age 46.6 63% female	To identify the key motivations of patients repeatedly seeking medical care for chronic back problems	Qualitative Questionnaires and interviews
Ong[43] 2011 UK	Duration of LBP not specified Duration ranged from <1 month to >3 years	37 participants	Purposive sampling of patients from the Keele BeBack patient study	Age range 19–59 59% female	To enhance the understanding of patients’ own perspectives on living with sciatica to inform improvements in care and treatment outcomes.	Qualitative Interviews
Rhodes[59] 1999 USA	People who had experienced LBP during the preceding 3 years. Episodes were defined as >1 visits for LBP spaced at least 90 days apart from any other visit for LBP.	54 participants	Interviews were conducted in 3 cities (Atlanta, Dallas and Seattle). Computerised databases used to identify eligible participants. Random sample of 110 patients were recruited.	Mean age 46.6 63% female	To explore the meaning of diagnostic tests for people with chronic back pain	Qualitative Interviews
Rogers[79] 1999 Australia	Duration of LBP not specified	21 GPs and 17 patients	Participants randomly recruited from an age and gender stratified list of GPs in a geographically defined region of South Australia	Age range 28–70 71% female	To study and report the attitudes of patients and GPs concerning the obligation of doctors to act for the good of their patients and to provide a practical account of beneficence in GP	Qualitative Semi-structured interviews
Sanders[37] 2015 UK	Duration of LBP not specified	37 participants	Purposive sampling of participants from 8 general practice settings	Average age not specified 60% female	To report patients’ changing experiences of back pain as shifting from a focus on incapacity, pain and physical limitation towards a more positive conception of illness which promotes patient empowerment	Qualitative Interviews
Scheermesser[74] 2012 Switzerland	Chronic LBP, duration not specified. Mean duration of LBP 7 years in men and 3.5 years in women.	13 participants	Participants were purposively sampled from the Rehabilitation Centre Clinic	Mean age 52 (men) and 48 (women) 31% female 3 from Serbia, 4 from Croatia, 3 from Bosnia, 1 from Macedonia and 1 from Kosovo (living in Switzerland mean 24.5 years in men and 16 years in women)	To identify what factors patients of Southeast European cultural background in multidisciplinary rehabilitation programs for LBP perceive to be important for acceptance or participation and are the patients’ perspectives similar to those of health professionals and scientific literature?	Qualitative Focus group and semi-structured in depth interviews
Schers[69] 2001 Netherlands	Acute LBP <6 weeks Subacute 6–12 weeks Chronic >12 weeks	31 GPs and 20 patients participated.	Purposive sampling of 40 general practitioners from a region in the eastern Netherlands. Each GP was asked to invite the first patient of >18yo with non-specific LBP.	Patients median age 43 (range 25–68) 45% female	To explore factors that determine non-adherence to the guidelines for LBP	Qualitative Semi-structured interviews
Sharma[58] 2003 USA	Duration of LBP not specified	1414 participated	Data derived from the baseline questionnaire of a prospective, longitudinal, non-randomised, practice-based observational study of patients who self-referred to medical doctors and doctors of chiropractic therapy.	MD–age 38.7 (10.83) and 52% female. DC–age 41.5 (11.68) and 52% female	To identify the salient determinants of patient choice between medical doctors and doctors of chiropractor for the treatment of LBP.	Quantitative Questionnaires
Skelton[25] 1996 UK	>1 recorded consultation for LBP	52 participants 12 participating GPs	1 general practitioner from 12 general practices was invited to recruit up to 7 consecutive patients presenting with LBP. A maximum of 6 patients per GP were interviewed.	Median age 45 (range 31–61) 50% female	To explore the views of patients about low back pain and its management in GP	Qualitative Semi-structured interviews
Slade[78] 2009 Australia	LBP > 8 weeks	18 participants	Recruitment by metropolitan and community newspaper advertisements and university email.	Mean age 51 (SD 10) 67% female	To determine participant experience of exercise programs for nonspecific chronic low back pain.	Qualitative Focus group discussion
Snelgrove[42] 2013 UK	Chronic LBP, duration not defined	10 participants	Purposive recruitment from a waiting list of patients referred to a medically led chronic pain clinic in the southern UK for assessment and possible treatment for unrelieved chronic LBP.	Age range 40–76 60% females	To gain a better understanding of living with chronic LBP.	Qualitative In depth interviews
Stisen[67] 2015 Denmark	Duration of LBP not specified	9 participants	Participants recruited from patients with acute conditions in a rheumatology inpatient ward	Average age 57 (range 26–83) 44% female	To investigate and develop an understanding of pain in patients with fear avoidance belief hospitalized for low back pain	Qualitative Interviews
Toye[41] 2012 UK	Persistent non specific LBP but duration not defined	20 participants	Patients with persistent nonspecific LBP attending a chronic pain management programme at 1 hospital between Jan and March 2005. Non-probability sampling of small groups of people.	Age range 29–67 65% females	To explore how patients with persistent LBP interpret and utilise the biopsychosocial model in the context of pain management.	Qualitative Semi-structured interviews
Wallace[57] 2009 USA	Chronic LBP (pain at the level of the waist or below). Chronic (daily pain and activity limitations nearly everyday for the previous 3 months or more than 24 episodes of pain that limited activity for 1 day or more in the previous year)	723 participants	Computed assisted representative telephone survey of individuals with chronic neck or LBP in North Carolina.	Mean age 54 (13.84) 66% female	To identify factors associated with patients’ satisfaction with their last health-care provider visit for chronic low back pain	Quantitative Questionnaires
Westmoreland[40] 2007 UK	Subacute or chronic neck or back pain but duration of pain not defined	20 participants	Purposive sampling of 20 participants with subacute or chronic neck or back pain were interviewed.	Age range 29–88 75% female	To explore patients’ views of receiving osteopathy in contrast with usual GP care, to provide insight into the psychological benefit of treatment, and to explore their views on how such a service should be provided and funded.	Qualitative Semi-structured interviews preceded by short questionnaires
Wilson[56] 2001 USA	LBP classified as chronic if patients reported they they had pain all the time Duration of LBP not specified	52 physicians from 8 states and 1137 patients. Of the 1137 patients, 522 had LBP 615 had respiratory problem	Substudy of a large initiative assessing the impact of radiological reimbursement policy change instituted by the United Mine Workers of America Health and Retirement Funds on radiology utilization Generalist Physicians (mostly rural) were asked to enrol 30 or more consecutive eligible patients by mail.	Mean age 54 (SD14) 54% females	To study patients presenting for outpatient treatment of respiratory problems and low back pain and to examine the magnitude of the effect of the patients’ perceived need for radiological studies on use of those services.	Quantitative Questionnaires
Yi[39] 2011 UK	Chronic LBP, duration not specified	124/414 agreed to participate	Participants with chronic LBP were identified from pain management clinics, community PT clinics and GP surgeries. Potential participants were contacted by the study team and sent questionnaires by post.	Age 20-34yo 8.1%, 35–49 40.7%, 50–64 37.4%, 65–79 12.2%, 80+ 1.6% 64% females	To investigate patient preferences for alternative pain management programs for managing chronic LBP in primary care.	Quantitative Questionnaires

The duration of back pain was either undefined or mixed in 39 (78%) studies[24,25,37–43,45–47,50,51,55–65,67–69,72–77,79–81,83,84] . While 11 (22%) studies reported on chronic back pain (>12 weeks duration)[44,48,49,52–54,66,70,71,78,82].there were no studies on acute back pain alone (<6 weeks duration).

There were 35 qualitative studies[25,37,38,40–46,48–54,59–61,65–69,71,74,75,77–79,81–84] with participant numbers ranging from 7 to 110, with a median of 23. There were 14 quantitative studies[24,39,47,55–58,62–64,70,72,73,76,80], with a median participant number of 628 (range 124–1555). Mixed methods were utilised in 1 study[70], which had 348 participants. A total of 10976 participants were included in this review. Of the 32 studies that presented summary statistics, the median age of the participants was 50 years with a female predominance (58% female).

### Quality of studies

**Fig 2:**
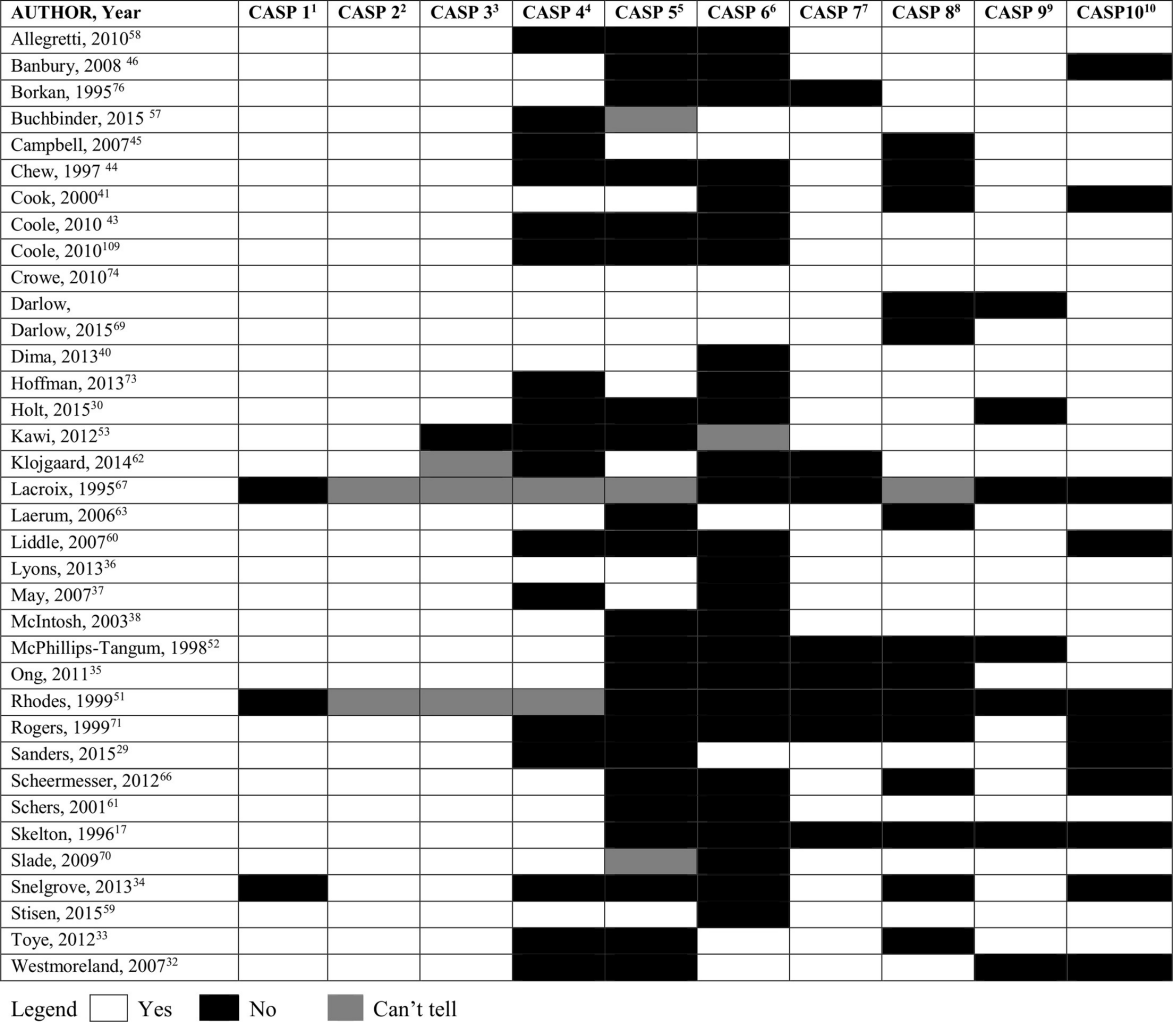
CASP tool for qualitative studies. ^1^CASP 1: Was there a clear statement of the aims of the research ^2^CASP 2: Is a qualitative methodology appropriate? ^3^CASP 3:Was the research design appropriate to address the aims of the research? ^4^CASP 4: Was the recruitment strategy appropriate to the aims of the research? ^5^CASP 5: Was the data collected in a way that addressed the research issue? ^6^CASP 6: Has the relationship between researcher and participants been adequately considered? ^7^CASP 7: Have ethical issues been taken into consideration? ^8^CASP 8: Was the data analysis sufficiently rigorous? ^9^CASP 9: Is there a clear statement of findings? ^10^CASP 10: How valuable is the research?

**Fig 3:**
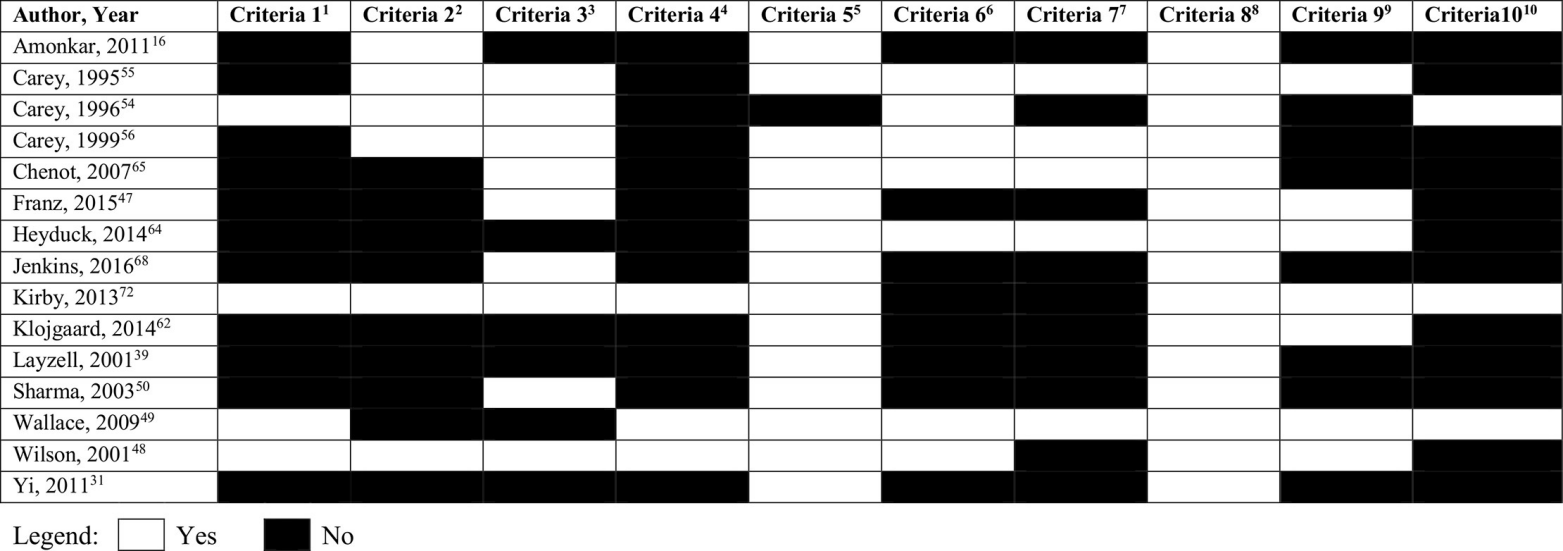
Hoy et al’s Risk of Bias tool for quantitative studies. ^1^Criteria 1:Was the study’s target population a close representation of the national population in relation to relevant variables? ^2^Criteria 2: Was the sampling frame a true or close representation of the target population? ^3^Criteria 3: Was some form of random selection used to select the sample OR was a census taken? ^4^Criteria 4: Was the likelihood of nonresponse bias minimal? ^5^Criteria 5: Were data collected directly from the subjects? ^6^Criteria 6: Was an acceptable case definition used in the study? ^7^Criteria 7: Was the study instrument that measured the parameter of interest shown to have validity and reliability? ^8^Criteria 8: Was the same mode of data collection used for all subjects? ^9^Criteria 9: Was the length of the shortest prevalence period for the parameter of interest appropriate? ^10^Criteria 10: Were the numerator(s) and denominator(s) for the parameter of interest appropriate.

Quality assessments of the included studies are presented in the Figs 2 and 3. The overall quality of qualitative studies was poor (Fig 2), especially for CASP criteria 4 to 6, indicating potential biases with data sampling and collection. The quantitative studies were of low quality: 10 studies were at high risk of bias, 4 studies were at moderate risk of bias and only 2 studies were at low risk of bias (Fig 3). The quality scores for both qualitative and quantitative studies largely reflected potential biases with recruitment strategy and data collection.

## Results of review

Four areas of perceived need were identified from the included studies (Tables 2–5).

**Table 2:** The perceived need for medical practitioners.

Author & Year	Results
***Role of the doctor and strengths of medical practitioners***	
Borkan 1995[84]	• Subjects wanted an exact diagnosis
Chenot 2007[73]	• 57% of patients seeing their GP were seeking additional specialist care.
Chew 1997[52]	• Subjects recognized that their GP was unable to help but viewed the doctor as a resource through which their social and economic inactivity could be legitimated
Coole 2010[50]	• Participants saw the main role of the GP was to prescribe medication, however many questioned the extent of its value
Coole 2010[51]	• Many patients thought there was little to be gained by consulting their GP and saw the main role of the GP as prescribing medication and providing sickness certificates
Crowe 2010[82]	• The majority of participants with chronic LBP had no regular contact with healthcare professionals, however 15 participants identified that healthcare professionals played a role in their self-management. The nominated professionals were predominantly physiotherapists or general practitioners.
Darlow 2013[83]	• Clinicians were seen as providing the most certainty, they could provide person-specific assessment and advice that participants hoped might prevent chronic LBP from developing
Hoffman 2013[81]	• Most believed in a biomedical approach (with the exception of analgesics) of needing to find the problem and fix it in a timely manner
Holt 2015[38]	• The clinicians’ provision of information and exclusion of serious disease were seen as helpful to patients, and helped them cope with their pain • Patients wanted a diagnosis, explanation of the cause of the pain and advice on how to manage the pain from their doctors
Kawi 2012[61]	• Patients felt that the primary role of the health care professional is to prescribe medications. They also thought that doctors should offer alternative modalities, including physical therapy, chiropractic, injections or interventional procedures.
Kirby 2013[80]	• GPs/specialists were the most common practitioner group consulted for pain relief (59.1%), followed by chiropractors (31.3%), PT (25.5%) and massage therapists (20.5%). • PT (31.7%) and chiropractors (30.4%) were the most common practitioner groups consulted for mobility improvement, followed by GPs/specialists (24%) and massage therapists (20.6%) • To improve function, women were more likely to consult with PT (23.9%) and chiropractors (23.9%) and GPs/specialists (20.0%) and massage therapists (16.7%). • GPs were the most common practitioner group consulted for general wellbeing (26.1%), followed by massage therapists (22.5%) and chiropractors (15.2%).
McPhillips-Tangum 1998[60]	• Nearly all participates described seeking medical care to discover the cause of their back problems • Most participants saw an increase in pain intensity or onset of pain as a signal to seek medical care
Ong 2011[43]	• Patients wanted a diagnosis from their doctor
Rhodes 1999[59]	• 98% of participants said that difficulty with normal activities drove them to seek care and 95% sought to discover the cause of their pain • Several patients held an attitude of joint expertise in which they recognized the importance and value of GPs medical knowledge but also discussed the significance of their own expertise in assessing their problem • Patients recognized medical knowledge and were glad to draw upon such expertise for the treatment of their problems yet these patients also saw an important role for less scientific knowledge
Rogers 1999[79]	• 95% of participants saw the GP to discover the cause of their pain
Sanders 2015 [37]	• Patients wanted reassurance from their doctor and they believed that the absence of a formal diagnosis (confirmed on x-ray or MRI) could mask a more “serious” pathology • Patients wanted a diagnosis and management options
Scheermesser 2012[74]	• Patients expect fast help, to be cured, healthy and pain free. They expected more pain-centred passive treatment (eg massage, hot packs, relaxation in the pool).
Schers 2001[69]	• Half of the patients reported that the main reason to visit the GP was to learn about the cause of symptoms and some patients expected to hear what they should do to improve and get rid of the symptoms. • Most patients expected to hear a specific diagnosis, although during the interview some could not remember hearing one. • Half the patients expected advice on desirable activities and half of the patients said that they were told to take it easy for a while.
Skelton 1996[25]	• 15/52 believe that it was appropriate to visit their GP routinely for episodes of LBP (of these 4 were primarily concerned about sickness certificates and the others saw such consultations as an opportunity to challenge misdiagnosis or inappropriate management or to explore alternative management strategies)
Slade 2009[85]	• Patients expect advice from practitioners and discussion of options for management.
Stisen 2015[67]	• Patients wanted a diagnosis or an explanation of the pain
Westmoreland 2006[40]	• GP strengths included continuity of care, listening and counseling skills
***Preference to see the doctor and satisfaction with the doctor***	
Amonkar 2011[24]	• 51% participants thought that specialist referral was valuable
Carey 1996[62]	• 61% of adults with acute severe LBP did not seek any health care during their most recent episode of pain however 24% initially sought care from a physician, 13% from a chiropractor and 2% sought care from other providers (physical therapist, nurse, massage therapist).
Carey 1995[63]	• Patients who saw orthopaedic surgeons where more satisfied than the patients who saw primary care providers but were less satisfied than those who saw chiropractors
Cook 2000[49]	• Participants frequently indicated an overwhelming faith in and dependence on doctors and the professions allied to medicine
Scheermesser 2012[74]	• 50% of patients would like to have seen their physician more frequently in rehabilitation programs
Toye 2012[41]	• Patients described the GP’s reluctance to refer to the specialist–they felt they had to make a strong case for their referral or the GP would ‘not sign that piece of paper’–this was described as a battle and some felt guilty for putting pressure on the doctor
***Inadequacies of the doctor***	
Borkan 1995[84]	• Physicians seen to have superficial approach and are mistrusted because of their tendency to delegitimize suffering and perceived as not taken seriously • Once certain pathological causes of LBP are eliminated, the physicians appear to slacken their investigations into the aetiology of the pain
Campbell 2007[53]	• Unmet expectations and inadequacy of medical doctors • Patients felt invalidated by medical model and perceived doctors as often just give medications
Coole 2010[51]	• Many perceived that there was little to be gained by consulting their GP about back pain • Some sought private investigations or physical therapy instead • Many participants reported that they had not received any advice or support in relation to work that they found effective • Little evidence of dialogue between GPs and other clinicians and employers, leaving the participants responsible for channeling and interpreting information between the 2 sectors
Layzell 2001[47]	• Felt there was a lack of knowledge on GPs party • Dissatisfaction in the way that back pain is viewed as each individual has specific problems
Liddle 2007[68]	• Treatment provided by GPs commonly referred to as being of little help in the long term with their primary emphasis being on the prescription of their pain killers and muscle relaxants
May 2007[45]	• Participants were dissatisfied with medical management, in particular, the protracted and ineffective episodes of care when tablets or rest were prescribed and the delay in referral to physiotherapy • Participants complained about the lack of empathy by specialists and the inappropriateness of what they had to offer.
McIntosh 2002[46]	• Patients felt that their GPs had not provided them with an ‘explicit’ diagnosis and none of the patients appeared to have any conception or understanding of the problem of diagnostic uncertainty in LBP. • Patients associate GPs’ perceived lack of diagnostic certainty with assumptions that the GP is either unable to help or believes them to be malingering and is thus withholding diagnostic information and access to more specialized back pain services.
McPhillips-Tangum 1998[60]	• Several patients expressed frustration over not receiving any diagnosis
Sanders 2015[37]	• Clinical explanations were perceived as inadequate, and back pain was presented as a common and “normal” problem with no clear options for addressing the problem
Toye 2012[41]	• Patients described how GPs lacked specialist knowledge that would allow them to effectively treat back
Westmoreland 2006[40]	• GP consulting time was perceived as restricted and therapeutic options limited or ineffective. • Many GPs attitudes were perceived as dismissive and patients felt disheartened and considered themselves a burden. • GP might be inadequately qualified in the complementary therapy and too tired or have insufficient time to provide an optimal service.
***Patients’ reasons for seeking medical care***	
Carey 1999[64]	• Patients with more severe levels of impairment were more likely to seek professional help for their symptoms
Rhodes 1999[59]	• 98% of participants said that difficulty with normal activities drove them to seek care and 95% sought to discover the cause of their pain
Sharma 2003[58]	Health status indicators associated with choice of MDs include greater pain, greater functional disability and chronic LBP. Patients who expected their care to be paid for by 3^rd^ parties were more likely to choose MD treatment when compared with self-pay patients

**Table 3:** The perceived need for pharmacological management.

Author, Year	Results
***Role of medications and patients’ preferences for medications***	
Amonkar 2011[24]	• Patient consider medications a slightly more useful option than doctors
Buchbinder 2015[65]	• Only 20% of patients in the study requested analgesics
Coole 2010[50]	• Patients were generally dismissive of medication as a treatment
Crowe 2010[82]	• A few of the participants used general practitioner-prescribed analgesics to manage their pain when it was severe. Most participants were generally resistant to taking medication regularly.
Dima 2013[48]	• Patients perceive medications as relaxing muscles, reducing inflammation, enabling detachment, provides temporary relief and prevents worsening, enables activity but use as a last resort.
Hoffman 2013[81]	• Some patients expected analgesics for the management of acute LBP.
Ong 2011[43]	• The perceived effectiveness of painkillers to deal with sciatica appeared to outweigh patients’ concerns about long-term consequences such as dependency. • Strong painkillers were needed to cope with daily life
Scheermesser 2012[74]	• Patients preferred passive treatments including medication, rest and did not understand why they should increase activity in the presence of pain, even though health professional seek to increase patients’ activity, coping and involvement.
Schers 2001[69]	• All patients said that they would take medications only if strictly necessary.
Stisen 2015 [67]	• Patients took pain killers to enable them to cope with social life
Toye 2012[41]	• All patients described the GP as ‘keen to dish out drugs’ but patients saw medication as just treating symptoms rather than ‘dealing with the actual problem’
Wallace 2009[57]	• Narcotic use was associated with satisfaction (OR 2.12, p = 0.01)
Yi 2011[39]	• Patients had a preference against education and medicines management, suggesting they do not consider medicines management to be an important part of a Pain Management Program
***Concerns regarding medications***	
Banbury 2008[54]	• Participants are generally confused about the value of complying with their analgesic regimen as healthcare professionals do not given them sufficient explanation when their prescriptions are issued.
Buchbinder 2015[65]	• Reluctance to request analgesics implies that patients perceive asking for analgesics to be a delicate and potentially stigmatizing act
Coole 2010[50]	• Many participants were uncertain about side-effects, effectiveness or the safety of the medication they had been offered and the impact on their work.
Dima 2013[48]	• Patients are concerned about side-effects, polypharmacy, addiction and desentisation, masks pain and could lead to further damage.
Lyons 2013[44]	• Many older adults reported they did not use their pain medication; some feared addiction and only took medicine, especially opioids when the pain became unbearable. Others reported S/E eg drowsiness.
Ong 2011[43]	• Patients did not like to impact of painkillers on sleep and that heavy sleep affected their mobility
Scheermesser 2012[74]	• Many patients felt trapped in vicious cycle of increasing pain and consumption of drugs
Snelgrove 2013[42]	• Reported a compounding dependence accompanied by a dislike of the deleterious side effects and growing lack of faith in medical treatments as the pain continued relatively unabated. • Participants’ lives were dominated by pain and medication.

**Table 4:** The perceived need for interventional therapies.

Author, Year	Results
***Preference for injections***	
Lyons 2013[44]	• Most avoided injections stating they would rather ‘live with pain’
***Preference for operations***	
Dima 2013[48]	• Patients feel that this is the last resort, medium term solution but are concerned about the inherent risks of surgery and implications for permanent changes to the spine.
Franz 2015[55]	• 52% of patients referred to a neurosurgery clinic would be willing to undergo surgery based on reported MRI abnormalities in the absence of symptoms • 33% of patients thought that surgery is more effective than physical therapy
Klojgaard 2014[70]	• Patients are willing to wait 2 years for the effects of treatment to avoid surgery
Lacroix 1995[75]	• “When one has constantly to take anti-inflammatory medication, there comes a moment when an operation becomes inevitable”
Lyons 2013[44]	• Most avoided surgery stating they would rather ‘live with pain’
***Characteristics of patients preferring surgery***	
Franz 2015[55]	• Men were more likely to believe that back surgery was more effective than physical therapy
Klojgaard 2014[70]	• Women are more reluctant than men to have surgery • Respondents who score highest on the pain scale are less willing to wait to avoid surgery • Patients with a shorter duration of pain were more willing to wait for avoid surgery, wait for better pain relief, improvement in performed ADLs • No differences with income, history of sick leave, expectations about results.

**Table 5:** The perceived need for imaging.

Author, Year	Results
***Preference for imaging***	
Amonkar 2011[24]	• >60% of participants thought that back x-rays were a positive investigation
Hoffman 2013[81]	• Most patients expected their GP to refer them for an X-ray particularly patients who felt that their pain was severe. Patients reported that the usefulness of x-ray outweigh the potential risks
Jenkins 2016[76]	• 54% of participants agreed or strongly agreed that radiological investigations are necessary to get the best medical care for low back pain • 48% of respondents agreed or strongly agreed that everyone with low back pain should have spine imaging
Schers 2001[69]	• Expectations on radiographic films varied. The patients who thought about radiographic film expected their GP to give in to their demands.
***Role of imaging***	
Allegretti 2010[66]	• Imaging that showed a physical defect seemed to provide closure for patients while a lack of definitive scan discouraged others
Darlow 2015[77]	• Patients felt stigmatized, as other people could not see their pain. Consequently, investigations are perceived to be very important to validate their experience
Holt 2015[38]	• Patients felt that they were being taking seriously when further investigations were being ordered by clinicians
Hoffman 2013[81]	• Many thought that an x-ray would enable the cause of the pain to be determined. • Patients felt that x-rays played an important role in providing reassurance as well as confirmation of their GPs diagnosis.
McPhilips-Tangum 1998[60]	• Minimisation of the seriousness of back pain by doctors, family and employers led some participants to seek a diagnostic test as a means to prove that some physical cause was underlying the pain
Rhodes 1999[59]	• 57% of participants talked about issues related to the need to egitimize their back pain and back condition and of these 28% talked about testing as an aspect of legitimation
Slade 2009[78]	• Ten participants expressed relief or an easier pathway when an x-ray or MRI demonstrated pathology.
***Characteristics of patients’ requesting imaging***	
Jenkins 2016[76]	• Increased age, lower education level, non-European or non-Anglosaxon cultural background, history of previous imaging and Back Beliefs Questionnaire scores were associated with beliefs that imaging was necessary

### The perceived need for medical practitioners (Table 2)

Twenty-three papers discussed the patients’ perceived role of the medical practitioner in the management of LBP[25,37,38,40,43,50,52,53,59,61,67,69,73,74,79–85]. A consistent theme that emerged from patients recruited from general practice [69,81,84,86], the community[43,60] and tertiary care was the need to obtain a diagnosis and a cause of the pain[37,38,59,60,67,69,79,81,84]. Other reasons for seeking medical care included a need to obtain medications for pain relief[50,51,61,80], to receive advice and discussion of options for LBP management[38,61,85], to receive sickness certification and legitimation of their back pain[25,51,52]. Patients also considered consultation with primary care medical practitioners as an opportunity to explore alternative medicines[25,61] and to obtain referrals to specialist medical or surgical services[73]. Patients generally viewed medical practitioners to be knowledgeable about their pain[53,79] and could provide individual assessment [83]. Westmoreland found that patients perceived the strengths of the medical practitioner to include continuity of care, listening and counselling skills[40].

Six studies identified factors related to patient preferences regarding the role of medical practitioners in LBP and their satisfaction with them[24,41,49,62,63,74]. Patients described having faith in medical practitioners and a dependence on them and professions allied to medicine[49]. Fifty-one percent of patients thought that specialist referral was valuable[24]. Patients have reported reluctance by the general practitioner to refer patients to a specialist[41]. A single study by Carey found that patients who saw orthopaedic surgeons reported higher satisfaction than those who saw primary health care providers[63].

Patients expressed their reasons for consultation with a medical practitioner in 3 studies[58,59,64]. It has been reported that 98% of patients sought medical care due to difficulty with normal activity and 95% of patients wanted to find the cause of their pain[59]. Patients with greater pain and more severe functional impairment were more likely to seek medical help for their symptoms[58,64].

Eleven studies reported on the patients’ perceived inadequacies of the medical practitioners[37,40,41,45–47,51,53,60,68,84]. Dissatisfaction with medical practitioners was reported from both qualitative[40,45,46,51,53,60,68,84,87,88] and quantitative[47] studies, as well as from all levels of care, including general practice[40,46,51,84,87], community-based[47,60], allied health clinics[45,47] and tertiary centres}[53,88]. Coole and Liddle found that patients felt there was little to be gained by consulting their primary care medical practitioner about their LBP[51,68] as they believed that they lacked specialist knowledge[41,46,47]. Patients felt that their consulting time with their medical practitioner was restricted and that therapeutic options were limited[40,45] and not individually tailored[47]. Furthermore, patients complained that medical practitioners had a cursory and superficial approach to the management of LBP, lacked empathy and had a tendency to be dismissive or delegitimise their symptoms[37,40,45,53,84]. Patients were disappointed that their medical practitioner did not provide a diagnosis[46,60] and they felt that the medical practitioner’s primary focus was on prescribing pain medications[53,68]. Also, patients were displeased with the delays in obtaining referrals to physiotherapy[45]. Patients also felt that once certain pathological causes of LBP were eliminated, medical practitioners appeared to slacken their investigations into the aetiology of pain[84].

### The perceived need for pharmacological management (Table 3)

Thirteen studies examined the need for medications[24,39,41,43,48,51,57,65,67,69,74,81,82]. Of these, 5 studies reported that patients preferred medications[24,43,57,74,81], and that analgesics enabled them to cope with their social life and activities of daily living[67]. Patients believed that medications would enable relaxation of muscles, reduce inflammation, provide pain relief, enable activity and prevent worsening of LBP[48]. Narcotic use was reported in 1 study to be associated with patient satisfaction[57]. However, Buchbinder found that only 20% of patients presenting to an academic Emergency Department with LBP requested analgesics, and those that did utilised strategies of mitigation, indirection and deference which suggested that they were aware of the intricacies of their requests[65]. Other studies of patients attending either rehabilitation or pain management programs found that the patients were generally dismissive of medication as a treatment[50] and felt that drugs were neither important nor appropriate in the management of LBP[39]. Furthermore, patients have described their general practitioners as being too “keen to dish out drugs” and patients viewed medication use as treating symptoms rather than managing the actual problem[41]. Some patients would only take medications if strictly necessary[69] and were generally resistant to taking medication regularly[82].

Patients recruited from all levels of healthcare (i.e. general practice, the community, specialist referral centres and allied health practitioners) have concerns regarding medications, which were reported in 8 studies[42,44,48,54,65,74]. Patients were apprehensive about the side-effects of medications and the potential for addiction and desensitisation[42–44,48]. Many patients felt trapped in a vicious cycle of increasing pain and consumption of drugs[42,74]. They were also concerned about the impact of medications on their work[51]. Furthermore, patients have reported confusion about medications and a lack of explanation by their healthcare provider[54]. Patients also expressed a reluctance to request analgesics for fear of stigmatisation, and if they did request medications, they were more likely to do so indirectly, particularly opioid-based analgesics[65].

### The perceived need for interventional therapies (Table 4)

Five studies explored patients’ preferences for interventional treatment for LBP[44,48,55,70,75]. A single study by Lyons assessed patients’ preferences for injection therapy and found that most patients avoided injections and would “rather live with the pain”[44]. Two studies reported that patients would rather avoid surgery and viewed surgical intervention as a last resort[44,48]. Franz found that half of the patients referred to a neurosurgical clinic were willing to undergo surgery in the absence of pain if they had radiological abnormalities, however, only 33% of patients believed surgery to be more effective than physical therapy[55]. Patients were willing to wait 2 years for the effects of conservative treatment to avoid surgery[70]. In comparison, Lacroix stated that patients felt that *“there comes a moment when an operation becomes inevitable”*[75]. Patients who preferred surgical intervention were more likely to be male, have higher pain scores and a longer duration of pain[55,70].

### The perceived need for imaging (Table 5)

Both qualitative and quantitative studies found that patients wanted imaging of their spine to find a diagnosis of their LBP[24,69,76,77,81]. Hoffman reported that most patients expected their general practitioner to refer them for an x-ray, particularly if they felt that their pain was severe[81]. Amonkar found that more than 60% of participants thought that back x-rays were a positive investigation[24]. Many patients felt that x-rays provided reassurance as well as confirmation of their general practitioner’s diagnosis[38,81]. Furthermore, imaging that showed a physical defect seemed to provide closure[66] and relief[85] for patients and patients sought diagnostic imaging as a means to legitimise their back pain[59,60,77].

Two studies examined the characteristics of patients requesting spinal imaging[56,76]. Wilson found that radiology utilisation was associated with the severity of back pain and a history of osteoporosis[56]. Jenkins reported that increased age, lower education level, non-European cultural background, history of previous spinal imaging and negative beliefs about back pain were associated with a perceived need for imaging[76].

## Discussion

This review identified 50 relevant articles that explored patients’ perceived needs for medical services for LBP. Four main areas of perceived need emerged, related to the need for (1) medical practitioners, (2) pharmacotherapy, (3) interventional therapies and (4) diagnostic evaluation. Patients with LBP sought healthcare from medical practitioners to obtain a diagnosis, sickness certification and to receive management options. However, patients were dissatisfied with a biomedical approach to care provided by medical practitioners. Patients saw a need for pharmacotherapy in pain management to facilitate function, however, they had concerns about medication side-effects and a fear of stigmatisation. Of the limited studies that examined the patients’ perceived need for invasive therapies, they reported that patients tend to avoid these treatment modalities. Furthermore, patients had misplaced beliefs about the necessity of imaging, and desired spinal imaging for diagnostic purposes and legitimation of symptoms.

Patients perceive a need for medical practitioners to obtain a diagnosis and strategies to cope with LBP and the associated disability[89]^,^[25,37,38,50,51,59–61,67,69,74,79–85]. In particular, patients with greater pain and more severe functional limitation sought medical help[58,59,64], thus highlighting the urgent need for more comprehensive and targeted delivery of effective and tailored pain management and coping strategies. In particular, it reinforces the importance of educating patients that in more than 90% of cases LBP cannot be attributed to a pathoanatomic cause, and is thus termed ‘non-specific’. Here, it is critical to reassure patients about their presentation and prognosis. [90]. The patients’ utilisation of medical services for sickness certification and legitimisation of their back pain has also clearly emanated from this review[25,43,50–52,60,69,79,84]. This mirrors the complexity of LBP and the widespread impact of the condition on social functioning, financial security and workplace satisfaction.

Patients have areas of dissatisfaction with the medical approach to management of LBP. They have expressed a lack of confidence in general practitioners in the management of their LBP[41,46,47], which may reflect the knowledge gap in primary care settings in LBP management[91,92]. This reinforces the need for training medical practitioners and further targeted education campaigns to upskill clinicians[93,94]. Patients were also displeased with the biomedically-focussed and cursory approach of medical practitioners in managing LBP[37,40,41,45–47,51,53,60,84]. This frustration with medical practitioners may stem from the biomedical paradigm used by many healthcare providers, which does not adequately consider the important psychological and social drivers to a pain experience nor address the patients’ need for holistic care[95]. Importantly, reliance on a biomedical approach to diagnosis and care in low back pain presentations is now considered overly reductionist and discordant with contemporary pain science. There is emerging evidence supporting the implementation of tailored therapy, addressing not only the physical aspects but also psychological factors in healthcare delivery for people with chronic LBP: this has been shown to improve health outcomes[96,97]. Despite a body of evidence supporting the biopsychosocial paradigm, practitioners encounter challenges in executing this approach to care[91,98]. In recent years, musculoskeletal Models of Care have been introduced[99–101]. These provide evidence-informed strategies for the delivery of patient-centred healthcare, including multidisciplinary pain management clinics, community-based education groups for patients, self-management group and individual programs for patients and carers, and education programs for primary care physicians. These interventions have been shown to improve health outcomes in terms of service delivery, patient satisfaction and health costs[96]. Further research is required to improve their implementation, assess cost effectiveness and promote the long-term sustainability of these approaches to care.

There is a wide spectrum of patient perceived need for pharmacotherapy in the management of LBP. Their needs are in line with current recommendations, with due consideration of potential side effects which require careful monitoring[102–105]. This review found conflicting beliefs regarding pharmacotherapy amongst patients, with some expecting medications for LBP management[24,43,48,50,57,67,74,81], whereas others were concerned about medication side-effects and the potential for addiction and desensitisation[42–44,48,50,54,65,74]. There is a critical need to rationalise the utilisation of prescription medication for LBP[106] with the recent epidemic of prescription drug misuse, particularly in developed countries[107,108]. The excessive use of opioids is problematic as there is little evidence to support the use of opiates for longer than 12 weeks, there are significant risks of addiction and death[107,109], and substantial costs[110]. This highlights the need for more effective training of medical practitioners in pain management and counselling patients regarding the use of prescription analgesics. Additionally, widespread patient education programs informing patients about the potential risks of pharmacotherapy, particularly opioids, should be provided and may have positive behavioural consequences that can lower the risk of addiction and abuse related to prescription medications[111].

Although some patients perceive a need for invasive interventions to manage LBP, there is limited or inconclusive evidence to support its use[112,113]. In addition to rising costs of pharmacotherapy for LBP, the costs of interventional therapies such as epidural and facet joint injections, as well as spinal surgery have also risen substantially[7]. Despite the widespread use of interventional modalities, this review identified only five studies[44,48,55,70,75] that described patients’ perceived needs for these therapies. These found that patients wanted to avoid interventional therapies such as injections and surgery[44,48,70]. Patients who preferred invasive interventions were more likely to be male, have higher pain levels and a longer duration of symptoms[55,70]. The relationship between their preferences and understanding of the risks and benefits of these procedures was not reported. These studies mainly recruited patients from hospitals, general practices or chiropractic clinics, thus representing a population of patients that have actively sought care for the management of their LBP, and potentially may have more disabling or persistent pain and are self-selected for a biomedically-oriented belief system about the aetiology of their pain. Health system interventions may need to be introduced to limit access to these therapies that lack evidence of effectiveness. Patient education and pain multidisciplinary management programs which embrace a biopsychosocial approach to care may also be used to better equip patients with more appropriate coping strategies for pain and address the patients’ perceived needs for interventional therapies in community-based populations[114].

Finally, many studies found that patients with LBP wanted imaging of their spine[24,69,76,77,81], despite the evidence-based recommendations to limit the use of radiological imaging[6–10,115], which is inappropriately overused[8]. Patients reported a preference for imaging to find a diagnosis, and some requested imaging to legitimise their back pain[24,38,59,60,66,76–78,81]. Patients’ preference for imaging suggests the need for additional public education about the inability to link the experience of pain with a structural pathology in the majority of cases[8,116] and appropriate utilisation of radiology and management of LBP. Public education campaigns have been used to reduce unnecessary radiology imaging[117], which may decrease the enormous economic burden of LBP. Addressing patients’ expectations and perceived needs of radiology utilisation may improve the provider-patient relationship, thus, improving health outcomes.

The results of this review need to be interpreted in light of a number of limitations. First, the included studies were heterogeneous in their study aims, study populations, participant sources, study design and methodology, thus the results of this study need to be interpreted in the context of heterogeneity in source data used. A further limitation of the design of the review is that potentially important differences between studies (e.g. population groups, healthcare settings) may be hidden by virtue of the analysis and reporting method used. Moreover, study populations were predominantly female. Participants were recruited mainly from hospital settings or general practices, rather than from the community. Additionally, many studies were from developed, English-speaking countries. These limitations restrict the generalizability of the results. Furthermore, few studies examined the possible effects of demographic variables such as age, gender, ethnicity, socioeconomic status, other co-morbidities and education on the perceived needs of medical services for LBP. Future studies examining specific subgroups defined by key characterising variables would be informative. Many of the included studies were susceptible to bias and had methodological limitations. However, as this was a scoping review, the main concern relates to a failure to capture populations that were not included and the breadth of perceived needs. Another limitation of this review is that there were no studies that specifically examined patients with acute LBP. Patients with acute LBP may have different perceived needs compared to those with chronic LBP, however, these were not differentiated in the primary papers we retrieved for this review. Therefore, the results from this review cannot be extrapolated to those with acute presentations of LBP. Future studies examining patients’ perceived need for medical services for acute LBP are warranted. Despite these limitations, this review incorporates qualitative and quantitative studies and encompassed four complementary databases to capture the breadth of the topic, and found consistent themes regardless of differences in study populations and methodologies. The data from studies was collated to provide an inclusive and in-depth description of the patient perspective of the medical management of LBP.

Patient expectations inform their use of and satisfaction with healthcare, particularly with conditions driven by symptoms, such as LBP. This review has highlighted the patients’ perceived needs and perceptions of the medical management of LBP and outlined gaps in our current knowledge, as well as areas of mismatch between patients’ perceived needs and evidence-based practice. The National Institute of Health and Care Excellence (NICE) guidelines for LBP acknowledge the importance of “tak(ing) into account the person’s expectations and preferences” in the implementation of evidence-based practice[118]. Moving forward, when formulating clinical practice recommendations, clinicians and guideline panels should collaborate with patient groups, to ensure incorporation of the patient perspective[119]. This may be achieved through a combination of consumer-centred Models of Care, public community education campaigns and enhancing clinicians’ communication skills to convey the appropriate messages. A coordinated educational campaign is required to bring medical management and patient expectations in line with evidence-based practice to optimize patient and health service outcomes.

## Supporting information

S1 File Search strategy. (DOCX)

S2 File PRISMA checklist. (DOCX)
