# Supplementary material for: Patients’ perceived needs for medical services for non-specific low back pain: A systematic scoping review
Source: PLoS One. 2018 Nov 8;13(11):e0204885. doi: 10.1371/journal.pone.0204885 (PMC6224057; doi:10.1371/journal.pone.0204885)
Supplement: S1 File — (DOCX) [file pone.0204885.s001.docx]

**S1 File – Search Strategy**

Database: Ovid MEDLINE(R) 1946 to Present with Daily Update

Search Strategy:

--------------------------------------------------------------------------------

1 exp Back Pain/

2 exp Low Back Pain/

3 low back pain.tw.

4 backache.tw.

5 back pain.tw.

6 backpain.tw.

7 coccyx.tw.

8 coccydynia.tw.

9 dorsalgia.tw.

10 (lumbar adj3 pain).tw.

11 lumbago.tw.

12 sciatica.tw.

13 sciatic neuropathy/

14 sciatica/

15 spondylosis.tw.

16 exp Spondylosis/

17 1 or 2 or 3 or 4 or 5 or 6 or 7 or 8 or 9 or 10 or 11 or 12 or 13 or 14 or 15 or 16

18 1 or 2 or 3 or 4 or 5 or 6 or 7 or 8 or 9 or 10 or 11 or 12 or 13 or 14 or 15 or 16

***************************

Database: Ovid MEDLINE(R) 1946 to Present with Daily Update

Search Strategy:

--------------------------------------------------------------------------------

1 (consumer* or patient* or client* or customer* or service user*).tw.

2 patients/ or inpatients/ or outpatients/

3 1 or 2

4 (rheumatolog* or doctor* or physician* or practitioner* or clinician* or specialist* or consultant* or health professional* or nurs* or allied health or physiotherap* or physical therap* or chiropract* or occupational therap* or podiatr* or nutrition* or diet* or rehabilitat* or pain management).tw.

5 health personnel/ or allied health personnel/ or nutritionists/ or physical therapist assistants/ or physical therapists/ or exp medical staff/ or exp nurses/ or exp physicians/

6 Rheumatology/

7 Manipulation, Chiropractic/ or Chiropractic/

8 nutrition therapy/ or diet therapy/ or caloric restriction/ or diet, carbohydrate-restricted/ or diet, fat-restricted/ or diet, reducing/

9 Counseling/

10 Psychology/

11 Dietetics/

12 Podiatry/

13 Rehabilitation Nursing/

14 Nursing Care/

15 Rehabilitation/

16 Pain Management/

17 ((conservative or surgical or orthop?edic or complementary or traditional or ayurvedic or acupuncture or chinese or herbal or moxibustion or homeopath*) adj3 (medicine* or therap* or treatment* or management)).tw.

18 complementary therapies/ or acupuncture therapy/ or acupuncture analgesia/ or moxibustion/ or homeopathy/ or medicine, traditional/ or medicine, chinese traditional/

19 ((exercis* or hyperthermia induc* or short wave or ultra* or ambulatory or rehab* or self help or electr* or manipulat* or manual* or heat) adj5 (therap* or modalit* or treatment*)).tw.

20 physical therapy modalities/ or electric stimulation therapy/ or exercise therapy/ or hyperthermia, induced/ or short-wave therapy/ or ultrasonic therapy/

21 "Physical and Rehabilitation Medicine"/

22 (tens or transcutaneous electric nerve stimulation).tw.

23 transcutaneous electric nerve stimulation/

24 (stretch* or strength* or mobili*).tw.

25 muscle stretching exercises/ or resistance training/

26 Manipulation, Orthopedic/

27 Musculoskeletal Manipulations/

28 ((joint* or knee* or hip*) adj3 (replac* or prosthe*)).tw.

29 (arthroplast* or hemiarthroplast*).tw.

30 arthroplasty/ or arthroplasty, replacement/ or arthroplasty, replacement, hip/ or arthroplasty, replacement, knee/ or hemiarthroplasty/ or arthroscopy/

31 ((anti-inflammatory or antiinflammatory or analgesic) adj3 (agent* or drug* or medic*)).tw.

32 ((nonsteroid* anti-inflammatory or nonsteroid* antiinflammatory or non steroid* anti-inflammatory or non steroid* antiinflammatory) adj (agent* or drug* or medic*)).tw.

33 pain killer*.tw.

34 analgesics/ or analgesics, non-narcotic/ or acetaminophen/ or ibuprofen/ or exp anti-inflammatory agents, non-steroidal/ or analgesics, short-acting/

35 Analgesics, Opioid/

36 steroid*.tw.

37 Steroids/

38 Prednisolone/

39 (disease modifying anti rheumatic adj (agent* or drug* or medic*)).tw.

40 antirheumatic agents/ or azathioprine/ or chloroquine/ or gold sodium thiomalate/ or gold sodium thiosulfate/ or hydroxychloroquine/ or methotrexate/ or sulfasalazine/

41 Biological Products/

42 Tumor Necrosis Factors/

43 Tumor Necrosis Factor-alpha/

44 Interleukin 1 Receptor Antagonist Protein/

45 Infliximab.tw.

46 Etanercept.tw.

47 Certolizumab.tw.

48 Golimumab.tw.

49 Interleukin 1 inhibitor.tw.

50 Anakinra.tw.

51 Canakinumab.tw.

52 Interleukin 6.tw.

53 Tocilizumab.tw.

54 CD-20.tw.

55 Rituximab.tw.

56 Co-stimulatory blockade.tw.

57 Abatacept.tw.

58 biologic*.tw.

59 tnf.tw.

60 Diphosphonates/

61 Bisphosphonate*.tw.

62 Vitamin D/

63 Cholecalciferol/

64 vitamin D.tw.

65 Calcium/

66 Calcium.tw.

67 self-help devices/ or wheelchairs/

68 exp Dependent Ambulation/

69 canes/ or crutches/ or orthotic devices/ or braces/ or walkers/

70 (walking adj3 (cane* or frame* or aid*)).tw.

71 self help devices.tw.

72 assistive devices.tw.

73 or/4-72

74 (utili* or need* or seek* or retriev* or provid* or provision or source* or aid* or promot* or access* or demand* or insufficien* or deficit* or gap* or barrier* or enabler* or facilitat* or deliver* or implement* or manag* or coordinat*).tw.

75 Needs Assessment/ or "Health Services Needs and Demand"/ or Health Services Accessibility/

76 74 or 75

77 ((consumer* or patient* or client* or customer* or service user*) adj4 (need* or want* or like* or interest* or prefer* or satisf* or perspective* or experience* or attitude* or belief* or practice* or concern* or support* or participat* or advoca* or center* or centr* or orient* or focus* or empower* or expect* or opinion* or view* or perceive* or perception* or tailor* or bespoke or involv* or priorit* or control*)).tw.

78 "patient acceptance of health care"/ or patient preference/ or patient satisfaction/ or Patient-Centered Care/ or Health Knowledge, Attitudes, Practice/

79 77 or 78

80 ((household or out of pocket) adj3 expen*).tw.

81 "cost of illness"/ or health expenditures/ or exp "fees and charges"/

82 Waiting Lists/

83 Rural Health/ or Rural Population/

84 Urban Health/ or Urban Population/

85 Primary Health Care/

86 secondary care/ or tertiary healthcare/

87 Vulnerable Populations/

88 exp Culture/

89 communication barriers/

90 (cost* or fee* or charge* or expen* or wait* or time* or rural* or remote* or urban* or primary or secondary or tertiary or acute* or cultur* or communicat* or language* or linguistic*).tw.

91 80 or 81 or 82 or 83 or 84 or 85 or 86 or 87 or 88 or 89 or 90

92 3 and 73 and 76 and 79 and 91

93 78 and 92

***************************
