# Supplementary material for: Patients’ perceived needs for medical services for non-specific low back pain: A systematic scoping review
Source: PLoS One. 2018 Nov 8;13(11):e0204885. doi: 10.1371/journal.pone.0204885 (PMC6224057; doi:10.1371/journal.pone.0204885)
Supplement: S2 File — (DOCX) [file pone.0204885.s002.docx]

| **S2: PRISMA CHECKLIST** | | | |
| --- | --- | --- | --- |
|  |  |  |  |
| **SECTION** | **ITEM** | **PRISMA-ScR CHECKLIST ITEM** | **PAGE** |
| **Title** | 1 | Identify the report as a scoping review | 1 |
| **Abstract**  Structured summary | 2 | Provide a structured summary that includes (as applicable) background, objections, eligibility criteria, sources of evidence, charting methods, results and conclusions that relate the the review questions and objectives | 3 |
| **Introduction**  Rationale  Objectives | 3  4 | Describe the rationale for the review in the context of what is already known. Explain why the review questions/objectives lend themselves to a scoping review approach.  Provide an explicit statement of the questions and objectives being addressed with reference to their key elements (e.g. population or participants, concepts and context) or other relevant key elements used to conceptualise the review questions and/or objectives. | 5-6  6 |
| **Methods**  Protocol and registration  Eligibility criteria  Information sources  Search  Selection of sources of evidence  Data charting process  Data items  Critical appraisal of individual  sources of evidence  Summary measures  Synthesis of results  Risk of bias across studies  Additional analysis | 5  6  7  8  9  10  11  12  13  14  15  16 | Indicate whether a review protocol exists: state if and where it can be accessed and if available (e.g. a Web address), provide registration information, including the registration number.  Specify characteristics of the sources of evidence used as eligibility criteria (e.g. years considered, language and publication status) and provide a rationale.  Describe all information sources in the search (e.g. databases with dates of coverage and contact with authors to identify additional sources) as well as the date the most recent search was executed.  Present the full electronic search strategy for at least 1 database, including any limits used, such that it could be repeated.  State the process for selecting sources of evidence (i.e. screening and eligibility) included in the scoping review.  Describe the methods of charting data from the included sources of evidence (e.g. calibrated forms or forms that have been tested by the team before their use, and whether data charting was done independently or in duplicate) and any processes for obtaining and confirming data from investigators.  List and define all variables for which data were sought and any assumptions and simplifications made.  If done, provide a rationale for conducting a critical appraisal of included sources of evidence; describe the methods used and how this information was used in any data synthesis (if appropriate).  Not applicable for scoping reviews.  Describe the methods of handling and summarising the data that were charted.  Not applicable for scoping reviews.  Not applicable for scoping reviews. | N/A  7-8  7-8  S1  7-8  8-9  7-8  9  N/A  8-9  N/A  N/A |
| **Results**  Selection of sources of evidence  Characteristics of sources of  evidence  Critical appraisal within sources of  evidence  Results of individual sources of  evidence  Synthesis of results  Risk of bias across studies  Additional analyses | 17  18  19  20  21  22  23 | Give numbers of sources of evidence screened, accessed for eligibility and included in the review with reasons for exclusion at each stage, ideally using a flow diagram.  For each source of evidence, present characteristics for which data were charted and provide the citations.  If done, present data on critical appraisal of included sources of evidence (see item 12).  For each individual source of evidence, present the relevant data that were charted that relate to the review questions and objectives.  Summarise and/or present the charting results as they relate to the review questions and objectives.  Not applicable for scoping reviews.  Not applicable for scoping reviews. | 10, Figure 1  Table 1  Fig 2 and 3  12-16, Table 2  12-16  Fig 2 and 3  N/A |
| **Discussion**  Summary of evidence  Limitations  Conclusions | 24  25  26 | Summarise the main results (including an overview of concepts, themes and type of evidence available), link to the review question and objectives and consider the relevance to key groups.  Discuss the limitations of the scoping review process.  Provide a general interpretation of the results with respect to the review questions and objectives, as well as potential implications and/or next steps. | 17-21  21-22  22 |
| **Funding** | 27 | Describe the sources of funding for the included sources of evidence, as well as sources of funding for the scoping review. Describe the role of the funders of the scoping review. | 23 |
